# UNDERSTANDING GOAL SETTING AND BEHAVIOR CHANGE MECHANICS IN OLDER ADULT SITTING REDUCTION

**DOI:** 10.1093/geroni/igae098.4361

**Published:** 2024-12-31

**Authors:** Mikael Anne Greenwood-Hickman, David Arterburn, Julie Cooper, Bev Green, Erika Holden, Jennifer McClure, Dori Rosenberg

**Affiliations:** Kaiser Permanente Washington Health Research Institute, Seattle, Washington, United States; Kaiser Permanente Washington Health Research Institute, Seattle, Washington, United States; Kaiser Permanente Washington Health Research Institute, Seattle, Washington, United States; Kaiser Permanente Washington Health Research Institute, Seattle, Washington, United States; Kaiser Permanente Washington Health Research Institute, Seattle, Washington, United States; Kaiser Permanente Washington Health Research Institute, Seattle, Washington, United States; Kaiser Permanente Washington Health Research Institute, Seattle, Washington, United States

## Abstract

While sedentary behavior (SB) is highly prevalent in older adults, scant evidence documents how participants actionably change this behavior. We explored intervention fidelity, participant satisfaction, and the types of goals participants set based on their preferences within a 6-month randomized controlled trial of a SB intervention for adults aged ≥60 years that successfully reduced participant sitting. Coaches tracked goals for all participants, and 8% of visits were randomly reviewed and coded for fidelity using a structured template. At study completion, participants completed a brief satisfaction questionnaire. Goals data were qualitatively coded and grouped by reminder strategy and topic. Fidelity and satisfaction data were summarized using frequencies and mean (standard deviation). Intervention participants (N=140, mean age 69 [6.0], 62% women, 66% Non-Hispanic White) had high satisfaction (95% Satisfied or Very Satisfied), and 83% reported perceived improvements to health and/or daily functioning. Fidelity to intervention content and techniques was also high across coded intervention sessions (SMART goals set in 98% of sessions, 93% active problem solving with coach, 95% discussed specific sitting break reminder strategies). Participants primarily set sitting reduction goals leveraging outward (e.g., fitness band prompts, using a standing desk for certain tasks) and habit (e.g., adding standing to a meal, standing during daily chores) reminder strategies tailored to individual preferences and lifestyle. Using inner cues such as awareness of pain or stiffness were less common. Specific goals to reduce SB varied widely, suggesting tailored SB reduction intervention approaches are likely important, particularly for older adults with chronic conditions.

